# Venezuelan Equine Encephalitis Virus Infection in Nonhuman Primate, Guatemala, 2023

**DOI:** 10.3201/eid3102.241484

**Published:** 2025-02

**Authors:** Wendy K. Jo, Marta Piche-Ovares, Lincoln Carranza, Carlo Fischer, Sebastian Brünink, Laura Paul, Alejandro Morales, Fernando Martinez, Jan Felix Drexler

**Affiliations:** Charité–Universitätsmedizin Berlin, corporate member of Freie Universität Berlin and Humboldt-Universität zu Berlin, Institute of Virology, Berlin, Germany (W.K. Jo, M. Piche-Ovares, C. Fischer, S. Brünink, L. Paul, J.F. Drexler); Wildlife Rescue and Conservation Association, Flores, Guatemala (L. Carranza, A. Morales, F. Martinez); German Centre for Infection Research, associated partner site Charité, Berlin (W.K. Jo, J.F. Drexler)

**Keywords:** Venezuelan equine encephalitis, viruses, VEEV, subtype IE, enzootic, Guatemala, nonhuman primate, conservation ecology, zoonoses, meningitis/encephalitis

## Abstract

We isolated Venezuelan equine encephalitis virus (VEEV) subtype IE phylogenetically related to Gulf Coast strains in a spider monkey (*Ateles geoffroyi*) released from a rescue center in Guatemala. Serologic testing of 118 monkeys indicated no additional VEEV infections. Infection of a primate warrants intensified surveillance of VEEV transmission cycles in North America.

Venezuelan equine encephalitis virus (VEEV) is an alphavirus in the Americas that can cause febrile illness and severe disease, including encephalitis. In humans, overall case-fatality rates are <1% but higher in children; in horses, case-fatality rates are 50%–70% (https://www.woah.org/en/disease/venezuelan-equine-encephalitis). In the United States, VEEV is classified as a select agent because of its pathogenicity and aerosolization capacity (https://www.selectagents.gov/sat/list.htm). The transmission of the arthropodborne VEEV involves an epizootic cycle (antigenic subtypes IAB and IC), entailing higher numbers of human infections, and an enzootic cycle (the common transmission cycle for antigenic subtypes ID and IE), entailing sporadic human infections (*1*,*2*). The emergence of VEEV epizootics is poorly understood but might involve genetic exchanges from enzootic subtypes (*1*). VEEV subtype IE has been reported in Central America and Mexico since the 1960s (*3*). Subtype IE has been detected almost exclusively in mosquitoes and sentinel hamsters (*3*,*4*) but sporadically in horses and humans (*2*,*3*,*5*); the subtype has been associated with 2 epizootics in horses in Mexico in the 1990s (*6*). We report the detection and isolation of a VEEV IE strain in a healthy nonhuman primate (NHP) in Guatemala in 2023.

We investigated 211 animals from 22 species (orders Artiodactyla, Carnivora, Didelphimorphia, Pilosa, Primates, and Rodentia) (Appendix Figure 1). We collected plasma samples from animals living in the Wildlife Rescue and Conservation Association (ARCAS) (https://arcasguatemala.org), a nongovernment organization in Petén Department in northern Guatemala. Animals are brought to ARCAS after being seized by police or customs officials at roadblocks or local markets. All animals are tested against selected pathogens and quarantined for 2–3 months upon arrival to the center and usually stay for another few months before being released into the wild (Appendix). We extracted RNA from plasma samples and screened it for alphaviruses and flaviviruses by using broadly reactive reverse transcription PCR (RT-PCR) assays (Appendix). By using an alphavirus RT-PCR, we identified VEEV in 1 adult male spider monkey (*Ateles geoffroyi*), an endangered species occurring from southern Mexico to Panama. No sample was positive for flavivirus RNA. Viral load in plasma was 1.8 × 10^5^ VEEV RNA copies/mL, determined through real-time RT-PCR (Appendix). At 2 days postinfection, Vero E6 cells displayed a cytopathic effect and reached high concentrations of 1.4 × 10^10^ VEEV RNA copies/mL of supernatant. We applied deep sequencing (Appendix) to the virus isolate and obtained a near-complete genome (4.7 million reads, >50,000× mean depth of coverage), encompassing 11,477 nt and lacking only 12 nt in the 5′ untranslated region (GenBank accession no. PQ406672). The VEEV genome showed typical organization; the predicted regions encoded nonstructural protein genes (*nsP1*–*nsP4*) and structural protein genes (*capsid*, *E1–E3*, and *6K*). The VEEV from Guatemala had pairwise nucleotide sequence distances of 1.4%–7.4% with VEEV subtype IE sequences available in GenBank that mostly originated from Central America (Figure 1). We identified no amino acid exchange at position 117 of the E2 glycoprotein, which previously had been associated with a VEEV subtype IE infection outbreak in horses (*1*). Pressure analyses that identify pervasive and episodic selection showed no sign of adaptive mutation in the E2 glycoprotein (Appendix Figure 2), suggesting strong purifying selection that might limit adaptation to new hosts (*3*). Bayesian phylogenetic analysis showed that the NHP-associated virus grouped with viruses obtained from the 1960s circulating in the Gulf Coast across a ≈200–700 km distance (most recent common ancestor 1954) (Figure 2), suggesting a lack of surveillance and continued circulation of genetically closely related strains in North America.

**Figure 1:**
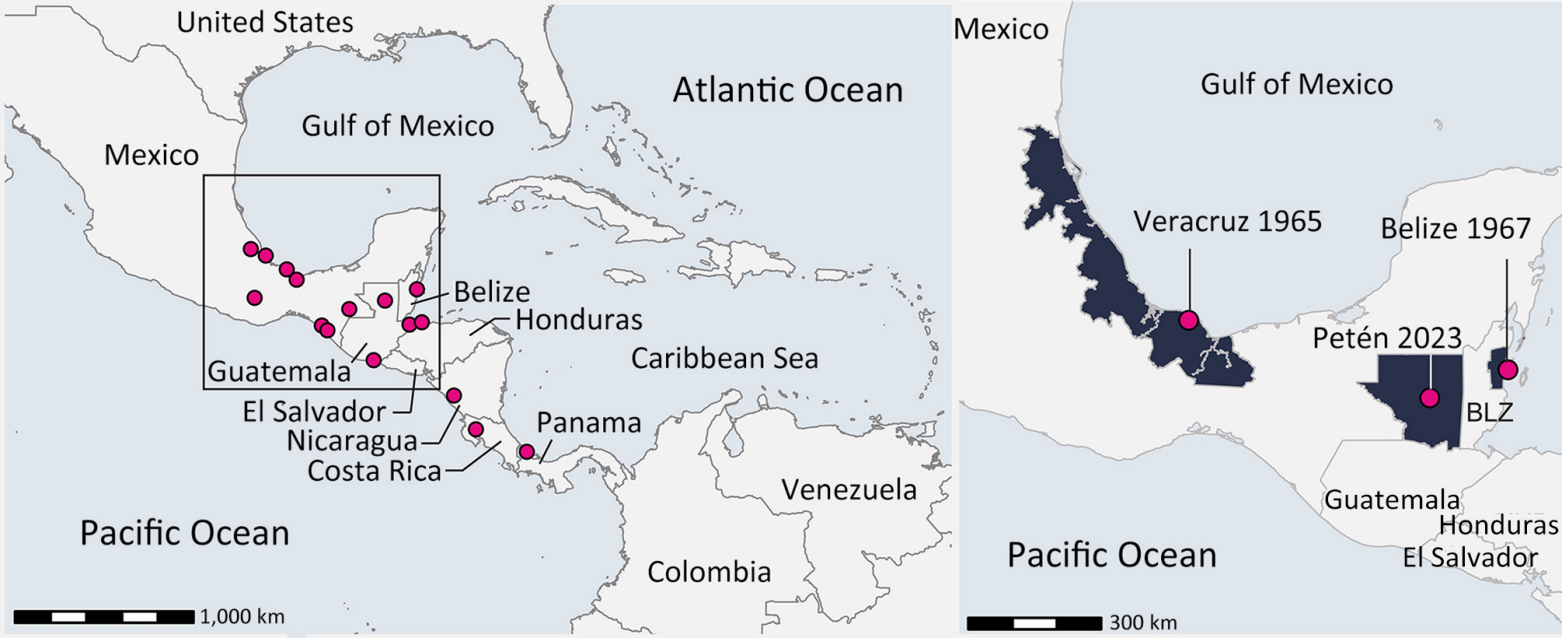
Geographic origins of reported Venezuelan equine encephalitis virus subtype IE sequences identified in Central America. Circles indicate geolocation of subtype IE sequences. Enlarged map shows the geolocation of the strains grouping with the virus from this study (Petén Department, Guatemala, 2023).

**Figure 2:**
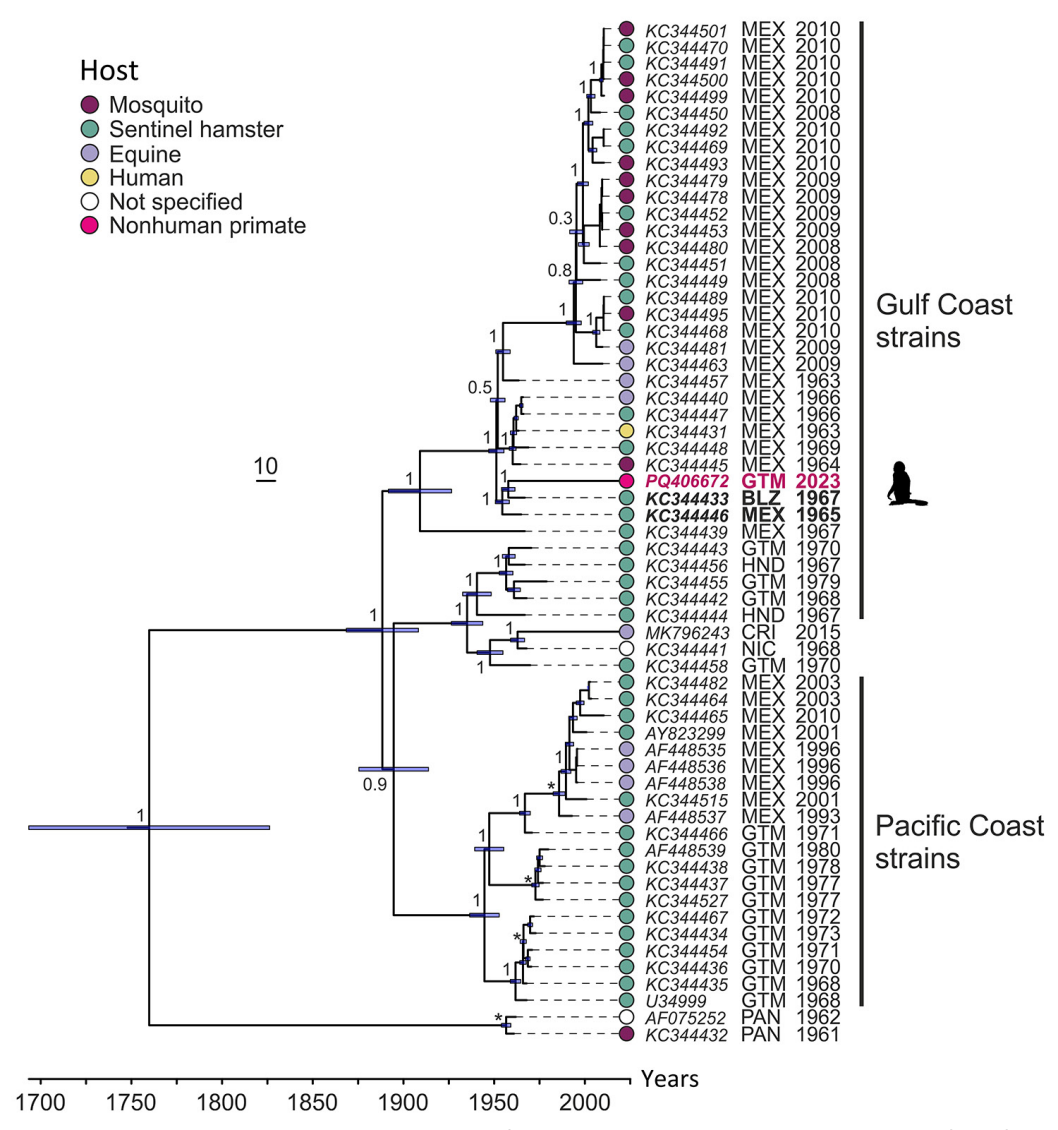
Time-scaled Bayesian maximum-clade credibility tree of Venezuelan equine encephalitis virus subtype IE identified in Central America. Bayesian phylogeny of the concatenated nonstructural and structural open reading frames with removal of the coding regions for the C-terminus of nonstructural protein 3 and N terminus of the capsid protein. Taxa indicate GenBank accession number, country abbreviation, and year of collection. Branch tips indicate host by color coding, including a sequence (strain no. 63Z1) isolated from blood of a sick human infected in the rainforest near Sontecamapan, Veracruz, Mexico, in August 1963 (*2*). Numbers at nodes indicate posterior probabilities of all major branches. Asterisks indicate clades previously used for dating according to a previous publication (*3*). Bars in node branches represent the 95% height posterior density intervals of the node ages. Scale bar represents time in years. BLZ, Belize; CRI, Costa Rica; GTM, Guatemala; HND, Honduras; MEX, Mexico; NIC, Nicaragua; PAN, Panama; SLV, El Salvador.

Natural VEEV infection in an NHP might be indicative of an outbreak. Therefore, we tested all plasma samples from primates residing in ARCAS (n = 118), including 80 spider monkeys and 38 howler monkeys (*Alouatta pigra* and *A. palliata*), for VEEV-specific IgM by using a modified commercial immunofluorescence assay (IFA) (Appendix). IFA detected no positive samples, including in the PCR-positive animal (Appendix Figure 3). The NHP infected with VEEV showed no clinical symptoms and was released into the wild on the day of blood sampling, suggesting that the animal was viremic upon release. In humans, severe disease characterized by neurologic complications occurs more frequently in children; therefore, future investigations might consider VEEV as a differential diagnosis, particularly in young NHP with acute neurologic disease in VEEV-endemic areas.

Although the neotropics are a probable hot spot for primate-associated emerging infections, neotropical NHPs are understudied for emerging pathogens (*7*). NHPs are among the most relevant sources of zoonotic viruses (in >20% of primate species zoonotic pathogens have been found) (*7*). Infection with other human pathogenic arthropodborne viruses, such as chikungunya and yellow fever viruses, has been reported in NHPs (*8*,*9*). Movement of NHPs by wildlife trafficking might contribute to the geographic expansion of VEEV and other pathogens. Although we identified no adaptive mutation in our study, the determinants of epizootics are not well understood, and new hosts might entail viral adaptation, potentially altering the viral phenotype. Seroepidemiologic and experimental infection studies, such as those conducted in rodents (*10*), are needed to clarify the role of NHPs in VEEV transmission cycles.

Appendix Additional information about Venezuelan equine encephalitis virus infection in nonhuman primate, Guatemala, 2023.
