# Supplementary material for: Venezuelan Equine Encephalitis Virus Infection in Nonhuman Primate, Guatemala, 2023
Source: Emerg Infect Dis. 2025 Feb;31(2):397–400. doi: 10.3201/eid3102.241484 (PMC11845163; doi:10.3201/eid3102.241484)
Supplement: Appendix — Additional information about Venezuelan equine encephalitis virus infection in nonhuman primate, Guatemala, 2023. [file 24-1484-Techapp-s1.pdf]

Article DOI: https://doi.org/10.3201/eid3102.241484

EID cannot ensure accessibility for supplementary materials supplied by authors. Readers who have difficulty accessing supplementary content should contact the authors for assistance.

Venezuelan Equine Encephalitis Virus Infection in Nonhuman Primate, Guatemala, 2023

Appendix

Additional Methods and Data

Sampling

Animals brought to the Wildlife Rescue and Conservation Association (ARCAS) in Guatemala are subjected to a protocol (Appendix Table).

After quarantine, animals go through different stations until they are ready to be released back into the wild. For this study, blood samples from live animals in ARCAS were collected in heparin tubes by trained veterinarians. Blood volume varied depending on the size of the animal. Blood was centrifuged at 4,500 rpm and plasma was collected and stored at −20°C until shipment. Shipping was conducted in dry ice and temperature was controlled (−84 – −60°C).

Virus detection

RNA was extracted from plasma samples using MagNA Pure 96 viral nucleic acid extraction kit (Roche Molecular Systems, USA). Samples were screened for flaviviruses and alphaviruses using broadly reactive RT–PCR assay as described previously (*1*,*2*). RT-PCR amplicons were Sanger sequenced (Microsynth Seqlab). Virus concentrations were evaluated using a RT-qPCR assay as previously described (*3*).

Immunofluorescence assay

Detection of VEEV IgM antibodies was performed using a commercial immunofluorescence assay (Catalogue No. FI 290a-1010 G, EUROIMMUN, Germany). The assay was adapted for IgM as the original assay is for IgG antibodies. Briefly, plasma samples, including the VEEV-PCR-positive sample, were pretreated with the immunoadsorbent Eurosorb

to deplete class IgM rheumatoid factors that can lead to false-positive results. Thereafter, pretreated samples are added to the slide followed by the addition of a fluorescein-labeled anti-human IgM, which allows cross-detection of antibodies from spider and howler monkeys as reported previously (*4*). All washes were performed with PBS-T. Immunofluorescence was visualized in a Leica DMi8 microscope.

Virus isolation

VEEV-positive sample was isolated in Vero E6 cells, cultured in DMEM media supplemented with 1% FBS, and 1% penicillin/streptomycin (PS). Cells were checked every day for cytopathic effect and supernatant was taken for evaluation of virus concentration by RT-qPCR as previously described (*3*).

Deep sequencing and genome retrieval

RNA library was prepared from the isolate according to the KAPA HyperPrep manufacturer's protocol (Roche) and sequenced on an Illumina MiniSeq system (150 cycles paired-end). Raw reads were quality trimmed, and a reference-based assembly to VEEV reference sequence (GenBank accession no. U34999) was then performed using Bowtie 2 (*5*).

Dataset

VEEV subtype IE sequences were searched for in the NCBI non-redundant database by blasting (BLASTn) our own VEEV sequence. A sequence identity of >90% was used as cutoff value and sequences belonging to subtype ID were removed from dataset. Sequences were retrieved from GenBank until 27/08/2024. The open reading frames (ORFs) 1 and 2 were concatenated and used for evolutionary analyses.

Evolutionary analyses

Translation alignments of the complete ORFs were performed with MAFFT (*6*) using the iterative refinement algorithm G-INS-i implemented within Geneious v11.1.5 (https://www.geneious.com). Pairwise nucleotide sequence distances were calculated using MEGA-11. For evolutionary analyses, the coding regions for the C-terminus of nsP3 and N terminus of the capsid were removed due to multiple inserts and deletions as previously described (*7*). Bayesian phylogenies were generated using the BEAST package V1.10.4 (*8*) with the substitution model LG+F+I+G4, an uncorrelated relaxed clock with lognormal distribution, and as tree prior the coalescent Bayesian skyline as previously described (*9*). Age priors were

included based on a previous VEEV subtype IE phylogenetic study (*9*). Age priors were assigned only to clades which included same taxa as in previous study (*9*). The analyses were run for 50 million generations with 10% burn-in, sampling every 1000 steps. A run was considered to have reached convergence when the effective sample sizes of all parameters were >200. Convergence was assessed in Tracer v.1.7.1 (https://beast.community/tracer). Selection pressure analyses of the E2 glycoprotein encoding region were done using the Bayesian method fast unconstrained Bayesian approximation (FUBAR) to infer pervasive selection (across the whole phylogeny) and mixed effects model of evolution (MEME) to infer episodic selection (at a subset of branches) implemented in the software package HyPhy (*10*).

References

1. Moureau G, Temmam S, Gonzalez JP, Charrel RN, Grard G, de Lamballerie X. A real-time RT-PCR method for the universal detection and identification of flaviviruses. Vector Borne Zoonotic Dis. 2007;7:467–77. PubMed

2. Grywna K, Kupfer B, Panning M, Drexler JF, Emmerich P, Drosten C, et al. Detection of all species of the genus Alphavirus by reverse transcription-PCR with diagnostic sensitivity. J Clin Microbiol. 2010;48:3386–7. PubMed https://doi.org/10.1128/JCM.00317-10

3. Carrera JP, Araúz D, Rojas A, Cardozo F, Stittleburg V, Morales Claro I, et al. Real-time RT-PCR for Venezuelan equine encephalitis complex, Madariaga, and Eastern equine encephalitis viruses: application in human and mosquito public health surveillance in Panama. J Clin Microbiol. 2023;61:e0015223. PubMed https://doi.org/10.1128/jcm.00152-23

4. Salas-Rojas M, de Oliveira-Filho EF, Almazán-Marín C, Rodas-Martínez AZ, Aguilar-Setién Á, Drexler JF. Serological evidence for potential yellow fever virus infection in non-human primates, southeastern Mexico. One Health Outlook. 2023;5:14. PubMed https://doi.org/10.1186/s42522-023-00090-5

5. Langmead B, Salzberg SL. Fast gapped-read alignment with Bowtie 2. Nat Methods. 2012;9:357–9. PubMed https://doi.org/10.1038/nmeth.1923

6. Katoh K, Standley DM. MAFFT multiple sequence alignment software version 7: improvements in performance and usability. Mol Biol Evol. 2013;30:772–80. PubMed https://doi.org/10.1093/molbev/mst010

7. Forrester NL, Palacios G, Tesh RB, Savji N, Guzman H, Sherman M, et al. Genome-scale phylogeny of the alphavirus genus suggests a marine origin. J Virol. 2012;86:2729–38. PubMed https://doi.org/10.1128/JVI.05591-11

8. Suchard MA, Lemey P, Baele G, Ayres DL, Drummond AJ, Rambaut A. Bayesian phylogenetic and phylodynamic data integration using BEAST 1.10. Virus Evol. 2018;4:vey016. PubMed https://doi.org/10.1093/ve/vey016

9. Forrester NL, Wertheim JO, Dugan VG, Auguste AJ, Lin D, Adams AP, et al. Evolution and spread of Venezuelan equine encephalitis complex alphavirus in the Americas. PLoS Negl Trop Dis. 2017;11:e0005693. PubMed https://doi.org/10.1371/journal.pntd.0005693

10. Pond SL, Frost SD, Muse SV. HyPhy: hypothesis testing using phylogenies. Bioinformatics. 2005;21:676–9. PubMed https://doi.org/10.1093/bioinformatics/bti079

Appendix Table. Protocol for animals brought to the Wildlife Rescue and Conservation Association (ARCAS) in Guatemala*

Animal order	Primates	Carnivora	Perissodactyla	Artiodactyla	Xenarthra
Quarantine days	90	60	90	90	60
Test					
Coproparasitic[I]	X	X	X	X	X
Tuberculosis[E]	X	–	X	X	–
COVID[E]	X	X	X	X	X
Malaria[E]	X	–	–	–	–
Dengue[E]	X	–	–	–	–
Chikungunya[E]	X	–	–	–	–
Zika[E]	X	–	–	–	–
Distemper[I]	–	X	–	–	–
Adenovirus[I]	–	X	–	–	–
Feline immunodeficiency[I]	–	X	–	–	–
Feline leukemia[I]	–	X	–	–	–
Parvovirus[I]	–	X	–	–	–
Canine coronavirus[I]	–	X	–	–	–
Giardia[I]	X	–	X	X	–
Entamoeba[E]	X	–	–	–	–
Cryptosporidium[E]	X	–	–	–	–
Toxoplasmosis[E]	X	–	–	–	–
Leishmania[I]	X	X	X	X	X
Anaplasma[I]	X	X	X	X	X
Ehrlichia[I]	X	X	X	X	X
Dirofilaria[I]	X	X	X	X	X

*I, internal test in ARCAS; E, external laboratory test

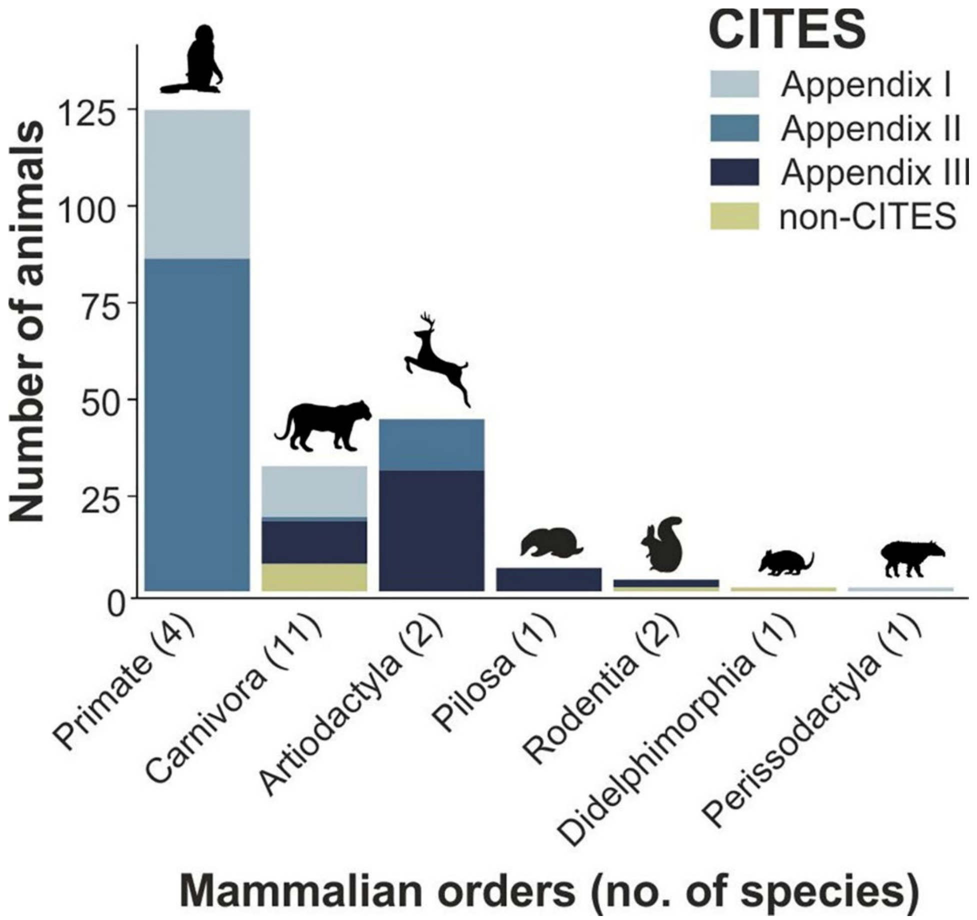

Appendix Figure 1. Distribution of investigated animals. Bars indicate individual animal counts per order. Number of species within each order are indicated in parenthesis. Color division represent the number of animals in each order that are enlisted in the Convention on International Trade in Endangered Species of Wild Fauna and Flora (CITES). Total animals = 211. All samples were collected from animals living in the Wildlife Rescue and Conservation Association (ARCAS), Flores, Petén, Guatemala.

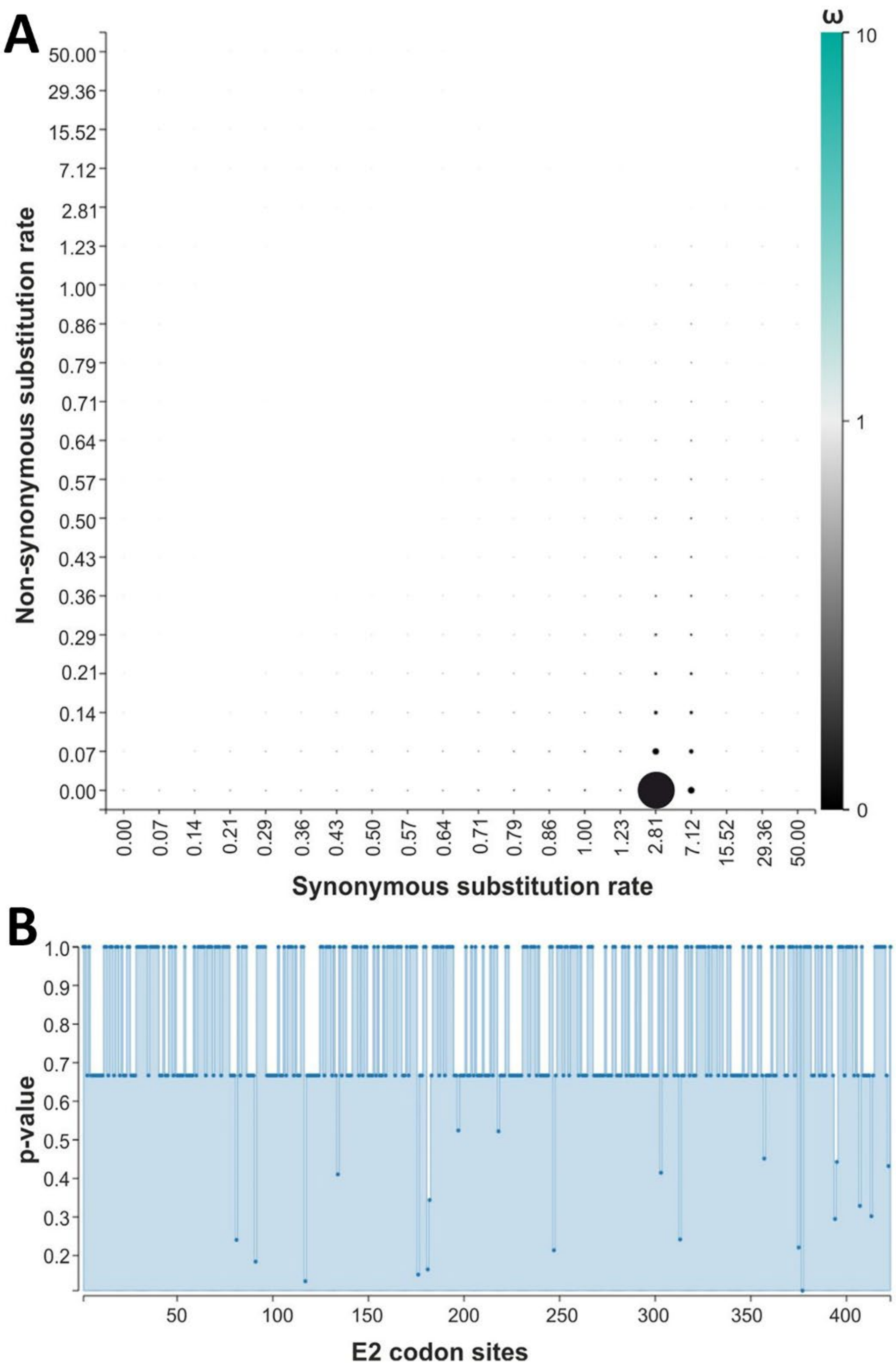

Appendix Figure 2. Pressure analyses. A) Graph representing FUBAR results showing posterior distribution over the discretized rate grid. The size of a dot is proportional to the posterior weight allocated to that gridpoint, and the color shows the intensity of selection. ω = nonsynonymous/synonymous rate. FUBAR found evidence of pervasive positive/diversifying selection at 0 sites, pervasive negative/purifying selection at 188 sites with posterior probability of 0.9. B) Graph representing MEME results showing p-value at the E2 codon sites. MEME found evidence of episodic positive/diversifying selection at 0 sites with p-value threshold of 0.1.

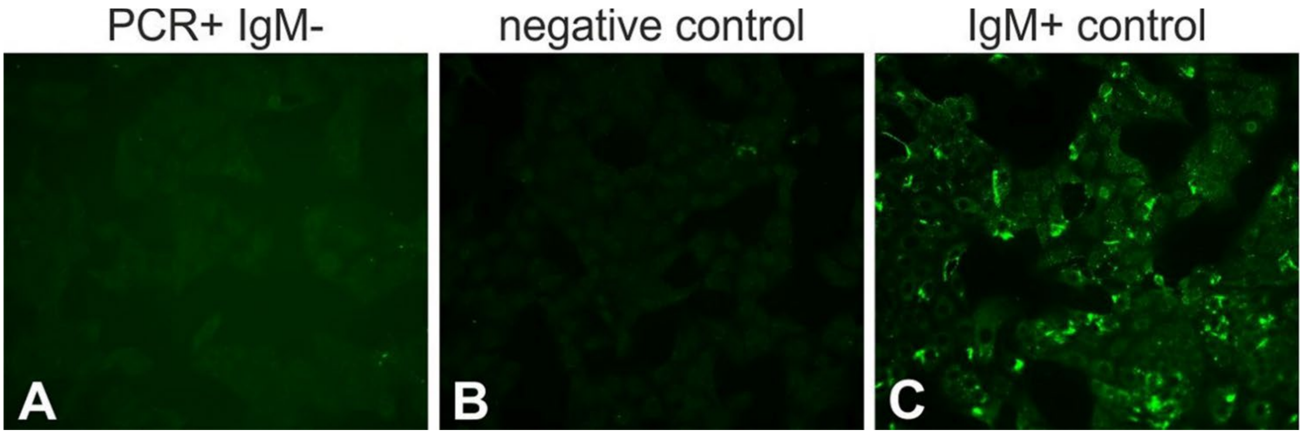

Appendix Figure 3. Venezuelan equine encephalitis virus immunofluorescence assay. Modified immunofluorescence assay (IFA; Catalogue No. FI 290a-1010 G, EUROIMMUN, Germany) for detection of VEEV IgM. A) Representative sample: spider monkey plasma (VEEV RT-PCR positive / infectious VEEV particles / IgM negative). B) Negative control (cells without VEEV antigen): spider monkey plasma in panel A. C) Positive control: human serum (VEEV IgM positive). All samples had a 1:10 dilution.
